# Empirical Evidence for Son-Killing X Chromosomes and the Operation of SA-Zygotic Drive

**DOI:** 10.1371/journal.pone.0023508

**Published:** 2011-08-17

**Authors:** Urban Friberg, Andrew D. Stewart, William R. Rice

**Affiliations:** 1 Department of Ecology, Evolution and Marine Biology, University of California Santa Barbara, Santa Barbara, California, United States of America; 2 Department of Evolutionary Biology, Uppsala University, Uppsala, Sweden; Ludwig-Maximilians-Universität München, Germany

## Abstract

**Background:**

Diploid organisms have two copies of all genes, but only one is carried by each haploid gamete and diploid offspring. This causes a fundamental genetic conflict over transmission rate between alternative alleles. Single genes, or gene clusters, only rarely code for the complex phenotypes needed to give them a transmission advantage (drive phenotype). However, all genes on a male's X and Y chromosomes co-segregate, allowing different sex-linked genes to code for different parts of the drive phenotype. Correspondingly, the well-characterized phenomenon of male gametic drive, occurring during haploid gametogenesis, is especially common on sex chromosomes. The new theory of sexually antagonistic zygotic drive of the sex chromosomes (SA-zygotic drive) extends the logic of gametic drive into the diploid phase of the lifecycle, whenever there is competition among siblings or harmful sib-sib mating. The X and Y are predicted to gain a transmission advantage by harming offspring of the sex that does not carry them.

**Results:**

Here we analyzed a mutant X-chromosome in *Drosophila simulans* that produced an excess of daughters when transmitted from males. We developed a series of tests to differentiate between gametic and SA-zygotic drive, and provide multiple lines of evidence that SA-zygotic drive is responsible for the sex ratio bias. Driving sires produce about 50% more surviving daughters than sons.

**Conclusion:**

Sex-ratio distortion due to genetic conflict has evolved via gametic drive and maternally transmitted endosymbionts. Our data indicate that sex chromosomes can also drive by harming the non-carrier sex of offspring.

## Introduction

Intragenomic conflict occurs whenever a new mutation has a selective advantage but causes reduced fitness of the organism as a whole or of another, non-allelic genomic component (e.g., cyto-nuclear conflict) [1], [2]. Burt and Trivers [3] have recently catalogued the surprisingly diverse forms of intragenomic conflict that occur in nature, examples of which include autosomal gametic drive with associated reduced fertility, sex chromosome gametic drive with associated sex ratio distortion, cytoplasmic endosymbionts that cause male sterility or sex reversal, homing endonucleases and transposable elements that increase the deleterious mutation rate, B chromosomes that reduce fertility, and maternal-effects coded by nuclear genes that kill those offspring that do not carry them. An additional category that we focus on here includes the male-killers [4]. The male-killing phenotype is produced by a wide diversity of cytoplasmic endosymbiotic bacteria that are propagated over successive generations only through the matriline. Male-killing leads to a selective advantage whenever *i)* there is sib-competition and the killing of sons/brothers frees up more shared resources for the daughters/sisters that propagate the endosymbiont, and/or *ii)* brothers mate with their sisters and thereby reduce the sisters' fitness due to inbreeding depression. This same logic can be applied to the sex chromosomes.

With male heterogamety, fathers transmit their Y chromosome to sons and their X chromosome to daughters. This sex-specific Mendelian segregation, when combined with sib-competition, generates natural selection for selfish X- and Y-linked mutations that harm the sex of offspring that does not carry them [5], [6], [7]. Such sexually antagonistic phenotypes *i)* reduce competition for shared resources among brothers and sisters carrying copies of their father's Y and X, respectively and *ii)* reduce the frequency of harmful sib-sib mating when there is inbreeding depression. The harmful, sex-specific phenotypes produced by these selfish mutations are collectively called ‘sexually antagonistic zygotic drive of the sex chromosomes’ (hereafter shortened to SA-zygotic drive).

The rationale for the occurrence of SA-zygotic drive is based on the logic of X/Y gametic drive, in which each type of sex chromosome is selected to disrupt the post-meiotic ontogeny of the type of sperm (X- or Y-bearing) that does not carry them. Competition between a father's X- and Y-bearing sperm in the haploid phase of the lifecycle leads to selection for the gametic drive phenotype. SA-zygotic drive extends this logic into the following diploid generation (driven by competition among diploid brothers [carrying the paternal Y] and sisters [carrying the paternal X]), where each sex chromosome is selected to disrupt the post-zygotic ontogeny of the sex of offspring that does not carry it. SA-zygotic drive is expected to evolve far more commonly, compared to offspring-harming selfish elements on the autosomes (like *Medea* elements in *Tribolium* [8] and the paternal killer *peel-1* in *Caenorhabditis elegans* [9]), because the sex-specific transmission of a father's X and Y chromosomes makes it relatively simple for sexually-antagonistic green-beard effect to evolve (as described in detail in Rice et al. [6]); a green beard effect occurs when a gene in some manner recognizes copies of itself in other individuals and responds in a way that increases the fitness of these individuals [1]).

SA-zygotic drive can be mediated by four major phenotypes [5], [6], [7], [10] *i)* paternal effects (or maternal effects when there is female heterogamety), through which a father's X and Y chromosomes are selected to harm offspring of the sex they are not transmitted to (especially in the context of trans-generational epigenetic influences on gene expression), *ii)* sib-sib interactions, because siblings of the same sex share the same paternal sex chromosome and therefore are predicted to exhibit more altruistic behaviours towards each other and more selfish/harmful behaviours towards opposite-sex siblings, *iii)* parent-offspring interactions, in which a father's X is selected to program him to favour daughters and harm sons, and vice versa for the father's Y, and *iv)* grandparent-grandchild interactions, where grandparents are selected to favour grandchildren that carry copies of their sex chromosomes and harm those that do not. A more complete description of the theory and its supporting evidence for SA-zygotic drive can be found elsewhere [6], [7], [10]. Previously we described published anthropological evidence supporting the conclusion that that SA-zygotic drive may be operating in humans via grandparent-offspring interactions [10]. Here we describe our search for SA-zygotic drive operating in a more experimentally tractable model organism.

The first lead in our search for an empirical example of SA-zygotic drive in a model system occurred when we (by the help of Hurst et al. [11]) located a short paper that described a failed attempt to document X-linked gametic drive in a laboratory stock of *Drosophila simulans* that had a female-biased sex ratio [12]. Negative results are commonly left unpublished but in this case were fortunately summarized and published in the non-refereed journal *Drosophila Information Service* (we detail the study in Appendix S1). In addition, the stocks studied were deposited at an international *Drosophila* stock center. Although these data did not confirm the operation of gametic drive, they were consistent with patterns predicted by SA-zygotic drive (see Appendix S1). The authors found a female-biased sex ratio that was associated with an elevated level of non-hatching eggs, the magnitude of which could fully account for the observed amount of sex ratio bias.

In the present study we develop a protocol to test for the operation of SA-zygotic drive. We then use this to show that the X chromosome, originally studied by Noor and Coyne [12], feasibly represents the first empirical example of SA-zygotic drive, operating via a paternal effect causing son-killing.

### A protocol to test for SA-zygotic drive

In flies with no male parental care, X driven SA-zygotic drive can occur via *i*) trans-generational epigenetic modification of an autosomal gene that exclusively harm sons by disrupting a male-specific developmental pathway that influences survival, *ii*) trans-generational epigenetic modification of the Y chromosome that disrupts (directly or indirectly via a regulatory effect on unlinked genes) any developmental pathway influencing survival, and *iii*) sib-sib interaction in which sisters harm the development of their brothers. All of these factors can lead to reduced survival of sons. We will use the term “trans-imprint” to refer to any epigenetic change coded by a sex chromosome that affects the expression or function of another part of the genome (an autosome or the other sex chromosome) and that persists, with probability >0, across at least one generation. This phenomenon includes any change in gene expression and is not restricted to the silencing of a maternally or paternally derived copy of a gene, as occurs in classic cases of genomic imprinting.

One can test for SA-zygotic drive by making an X chromosome substitution line of an SA-zygotic-driving X chromosome (X_skew_) into a line with a non-driving X chromosome (X_even_). Once constructed, the crosses shown in Table 1 can be made. The sons from these crosses have identical genotypes, cytotypes, and maternal effects. If the survival of sons from X_skew_/Y sires is reduced prior to or during sib competition, then there is ‘proof-of-potential’ evidence for SA-zygotic drive. Irrefutable evidence for SA-zygotic further requires that the observed X-coded sire-effect be sufficiently sex-specific to cause a female-biased sex ratio within broods.

**Table 1:** Crosses to produce genetically identical sons from fathers with different X-chromosomes.

Sires	Dams	Sons
X_skew_/Y ; A/A	X_even_/X_even_ ; A/A	X_even_/Y ; A/A
X_even_/Y ; A/A	X_even_/X_even_ ; A/A	X_even_/Y ; A/A

X_skew_ and X_even_ are X-chromosomes that produce a skewed and an even sex-ratio, respectively. Y stands for the Y-chromosome and each “A” stands for a haploid set of autosomes.

Full implementation of the assay described above requires that the sex of zygotes be determined so that all components of their embryonic and juvenile survival can be measured. Because this requirement can be difficult to achieve, even in model organisms, an indirect method can be accomplished by determining the sex of offspring at a point in time later than the zygote stage. In the experiments described here, we measured the number of eggs (E) laid by an inseminated female and then tallied the number of sons (M) and daughters (F) at the end of sib-competition (eclosion of adults). This approach can provide strong evidence for a pivotal paternal phenotype required to fuel SA-zygotic drive, but as described below, the evidence for the operation of SA-zygotic drive is probabilistic and not absolute.

**Figure 1:**
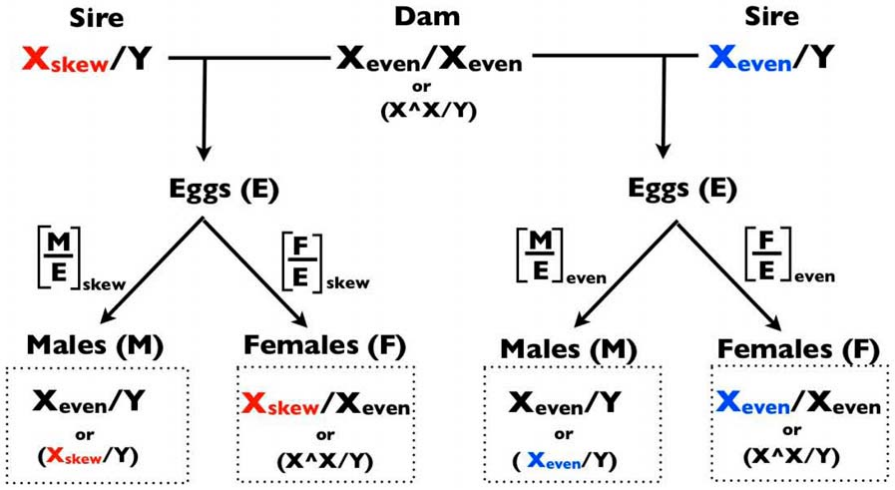
Crosses used in the experimental design. The transmission of sex chromosomes when X_skew_/Y and X_even_/Y sires are crossed to X/X and attached-X (parenthetical entries) dams. M/E and F/E denote the proportion of eggs that develop into mature male and female offspring, respectively.

**Figure 2:**
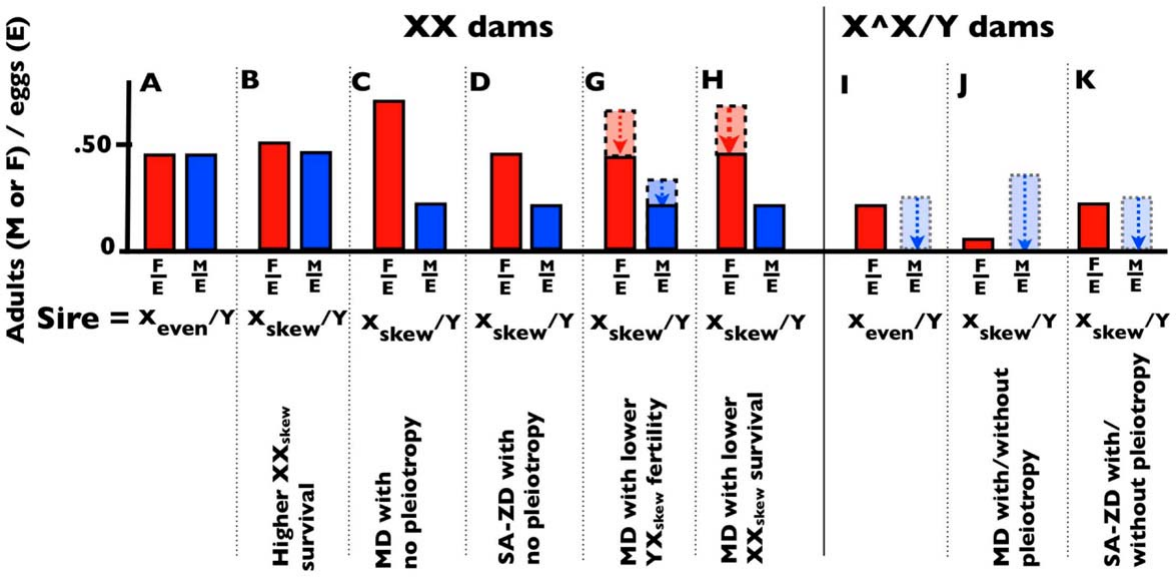
Contrasting expected patterns of egg-to-adult survival associated with X-linked gametic drive and SA-zygotic drive. Plotted are the expected proportion of eggs (E) developing into male (M/E) and female (F/E) offspring, measured at the end of sib-competition. **X/X dams:** (**A**) No sex ratio bias from Y/X_even_ sires (M/E≈F/E); (**B**) Increased survival of daughters expressing X_skew_; (**C**) Simple gametic drive when X_skew_ produces no pleiotropic influence on the sire's fertility nor the survival of his daughters; (**D**) Simple SA-zygotic drive with no pleiotropic influence of X_skew_ on the sire's fertility nor the survival of daughters. The pattern expected from simple SA-zygotic drive can also be produced by gametic drive when combined with a counterbalancing reduction in fertility of X_skew_/Y sires (**G**) and/or reduced survival of his daughters (**H**). Note that dotted arrows depict eggs not surviving to adulthood due to a sire's infertility or reduced viability of its carriers. **X∧X/Y dams:** When the dams carry an attached-X (Y-bearing sperm produce daughters), egg survival of sons and daughters from both types of sires is reduced by at least 50% due to the production of aneuploid zygotes (**I**), but gametic drive of the X will cause the expected value of F/E to be lower when the sire is X_skew_/Y, compared to X_even_/Y (**J**), and the sex ratio should be more male biased (unless the hemizygous expression of the two types of X chromosomes strongly influences male viability –dotted arrows). With SA-zygotic drive (**K**) the sex ratio from X_Skew_/Y sires should be unchanged compared to X_even_/Y sires (unless the Y chromosome is imprinted in a manner that harms females). With gametic drive, irrespective of any effects of the X_skew_ on fertility of sires or viability of daughters (assumed to be consistent across the two types of dams), the relative success of Y-bearing sperm (measured by the ratio of [F/E]_sire = skew_ to [F/E]_sire = even_ = *RS_Y(dams = X∧X)_*) from attached-X dams should equal that from X/X dams ([M/E]_sire = skew_ to [M/E]_sire = even_ = *RS_Y(dams = XX)_*).

Consider the two crosses of X_skew_/Y sires and X_even_/Y sires to X_even_/X_even_ females (Fig. 1), and assume that *i)* the sex ratios from these crosses are measured soon after sib competition (at eclosion in flies), and *ii)* the genetic backgrounds (Y, autosomes, and cytotypes) of both types of sires are the same. A father's X chromosome can contribute to a female-biased sex ratio of his offspring in several ways: *i)* increasing the survival of daughters that carry it (Fig. 2B), *ii)* reducing the production, survival (during development) or competitive ability of sperm (haploid) that do not carry it (gametic drive, Fig. 2C), *iii)* reducing the survival of developing siblings (diploid) that do not carry it (SA-zygotic drive, Fig. 2D), or *iv)* a combination of the three (e.g., Fig. 2G,H). Despite the multifarious forms of potential causation, the pattern shown in Fig. 2D (depressed value of males per egg [M/E], but no increase in females per egg [F/E], compared to the even sex-ratio cross) represents strong evidence for SA-zygotic drive when the operation of gametic drive can be ruled out (Fig. 2G,H).

The simplest way to assay for gametic drive is to cross X_skew_/Y sires to attached-X females (X∧X/Y). In *Drosophila*, an attached-X female carries two X chromosomes attached to a common centromere (X∧X) and a free Y chromosome, which unlike mammals, does not cause X∧X/Y zygotes to develop into males. X-bearing sperm from an X/Y male mated to an attached-X female produce X/Y sons and Y-bearing sperm produce X∧X/Y attached-X daughters, reversing the inheritance pattern of sex-chromosomes transmitted from males (Fig. 1). Half of the zygotes are nonviable due to aneuploidy (Y/Y of X/X∧X): Y/Y die as embryos and X/X∧X as larvae or pupae [13]. A simple assay for gametic drive is to cross X_skew_/Y males to attached-X females and test to see if the sex ratio bias is reversed (an excess of sons, Fig. 2I,J,K), compared to the one produced with normal X/X females. A negative result from this test provides strong support for the absence of gametic drive, but it is not fully convincing because low viability associated with the hemizygous expression of X_skew_ in sons could, fortuitously, counterbalance the sex ratio skew expected by gametic drive by reducing the number of surviving sons (Fig. 2J). The strongest support for SA-zygotic drive in this test would occur when both types of sires produce nearly the same sex ratio. A false negative in this case would require a nearly exact counterbalancing of the sex ratio skew of gametic drive by the viability effects of the two hemizygous X chromosomes.

A more convincing way to show that gametic drive is the factor contributing to sex ratio bias is to focus exclusively on the values of M/E (males/total eggs in broods) when the two types of sires are crossed to X/X dams and F/E (eclosing females/total eggs in broods), when the two types of sires are crossed to attached-X dams. These metrics measure the reproductive success (RS_Y_) of a sire's Y chromosome from the start of meiosis to the end of sib-competition, yet they are not influenced by any potential effects of X_even_ and X_skew_ on the viability of its carriers because sons from X/X dams have the same genotypes and cytotypes irrespective of the identity of the sire, as do daughters from attached-X dams. As shown in Appendix S2, when gametic drive is operating
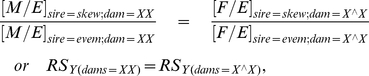
and as shown in Appendix S3, when SA-zygotic drive is operating
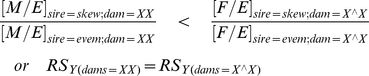
Although this test for the operation of gametic drive is robust to most effects that the X_skew_ and X_even_ chromosomes have on a sire's fertility, and any effect these chromosomes have on the survival of offspring that carry them, it can produce a false positive when the relative fertility of the two types of sires (proportion of dams' eggs fertilized by X_Skew_/Y sires compared to X_Even_/Y sires) changes with the different types of dams (XX and attached-X), i.e., when the fertility of X_even_ sires, relative to X_skew_ sires, is lower in attached-X dams (Appendix S2).

In summary, the pattern of egg-to-adult survival shown in Fig. 2D (reduced M/E but not F/E, compared to a cross with Y/X_even_ sires) provides strong evidence for the operation of SA-zygotic drive, but only when it can be shown that gametic drive is not operating. X-coded gametic drive can be assessed by looking for a reversed sex ratio (male-biased) when sires are crossed to attached-X dams –but strong survival effects of the hemizygous X chromosomes can potentially obscure this pattern. An additional test for gametic drive can be obtained by comparing measures of the relative success of Y-bearing sperm from the two types of sires (*RS_Y(dams = XX)_* vs. *RS_Y(dams = X∧X)_*). Deviation from equality of these two measures provides further evidence against the hypothesis that gametic drive is the sole agent responsible for a female-biased sex ratio, and finding *RS_Y(dams = XX)_*<*RS_Y(dams = X∧X)_* supports the conclusion that SA-Zygotic drive is operating. We will refer to the test based on the relative success of Y-bearing sperm as the XX/attached-X screen for SA-zygotic drive. Note that this set of tests also can be utilized to test for Y-linked SA-zygotic drive by reversing the sex of the offspring compared from the X/X and attached-X dams.

In this study, we apply the above protocol to X chromosomes taken from the two stocks studies by Noor and Coyne [12]. Chromosomal substitution lines (X_skew_/Y; A/A and X_even_/Y ; A/A) were produced by repeated backcrosses to the same inbred line containing females with an attached-X chromosome [C(1) *y*, *w*]. For each type of sire (X_skew_/Y and X_even_/Y), we measured the values of F/E and M/E within individual broods.

## Results

**Figure 3:**
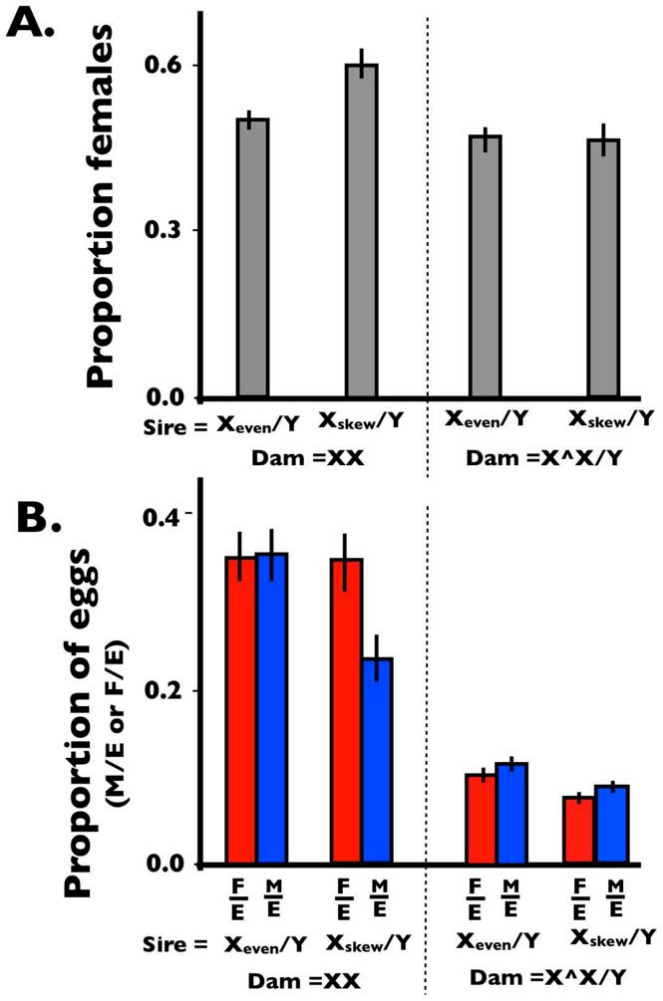
Assay results from test for SA-zygotic drive. (A) The observed sex ratio (expressed as proportion females) of newly emerging adults when X_skew_/Y or X_even_/Y sires were crossed to X/X or attached-X dams. (B) The observed proportion of males and females per total eggs laid (M/E or F/E) from dams (X/X or attached-X) mated to X_skew_/Y or X_even_ Y sires. Error bars represent 95% bootstrap confidence intervals.

When crossed to X/X females, the average proportion of females produced by X_even_/Y sires was 0.495 (95% CI = [0.478, 0.512]; Fig. 3A). When the sires were X_skew_/Y the proportion females averaged 0.596 (95% CI = [0.569, 0.623]; Fig. 3A). These non-overlapping CIs demonstrate that the X_skew_, when expressed in sires, was associated with a female-biased sex ratio. When X_even_/Y sires were crossed to attached-X females, the average proportion of females was 0.469 (95% CI = [0.446, 0.491]), and this value was 0.462 (95% CI = [0.433, 0.493]) when attached-X females mated to X_skew_/Y sires (Fig. 3A). Sex ratios somewhat less than 50% are common in attached-X crosses owing to the lower egg-to-adult viability associated with the females expressing the attached-X chromosome. The similar point estimates, and the strong overlap of their CIs, indicate that the sex ratio produced by both types of sires was highly similar when they were mated to attached-X dams: a result that does not support the operation of gametic drive, but is predicted by SA-zygotic drive. In contrast to our results, previous studies of X-linked gametic drive in *D. simulans* did observe a male biased sex ratio when sires carrying the driving X were crossed to attached-X dams [e.g., 14].

To look for the hallmark signature of SA-zygotic drive (Fig. 2D), we plotted the proportion of total eggs that survived to become adult males (M/E) or females (F/E) (Fig. 3B). As expected, when the dams were X/X, the values of F/E and M/E were very similar when the sires were X_even_/Y (mean and 95% CI for F/E and M/E were 0.351 [0.321, 0.379] and 0.357 [0.329, 0.385], respectively). These low values (<<0.5) were not unexpected since the even stock had a hatch rate of 69.7% (SE 0.02) after 48 h, which is considerably lower than the 91% hatch rate reported by Noor and Coyne [12]. When X/X dams were crossed with X_skew_/Y sires, the ratio F/E was similar to that from X_even_/Y sires (mean and 95% CI for F/E are 0.343 [0.310, 0.376] but that for M/E was significantly (non-overlapping 95% CIs) depressed (mean and 95% CI for M/E 0.233 [0.207, 0.258]; Fig. 3B). This pattern was seen in each of the three backcross generations (data not shown). The fact that the female biased sex ratio produced by X_skew_/Y sires was associated with a lower value of M/E compared to families from X_even_/Y sires, but a similar value of F/E from both types of sires (i.e., there was no indication that daughters from X_skew_/Y sires had elevated survival), supports the conclusion that pleiotropic viability effects of the X_skew_ chromosome on its carrier were not the responsible for the observed female-biased sex ratio. Sons from both sires had the same average genotypes and maternal effects, and thus should have similar survival, especially under the low density conditions under which the progeny were reared (an average of <20 larvae per vial), yet the reduced value of [M/E]_skew_ accounts for all of the sex ratio bias from X_skew_/Y sires.

When X_even_/Y sires were crossed to attached-X females (Fig. 3B), both F/E (mean and 95% CI are 0.106 [0.098, 0.115]) and M/E (mean and 95% CI for are 0.1205 [0.112 0.129]) were a small amount higher than when X_skew_/Y sires were mated to these females (means and 95% CE for F/E are 0.081 [0.074, 0.088] and for M/E are 0.094 [0.085, 0.103]). This same pattern was observed in each of the 4 backcross generations (data not shown). These data suggest that X_skew_/Y sires may have somewhat lower fertility than X_even_/Y sires, but the similar sex ratio for both types of males does not support the operation of gametic drive. All of the results from this and the previous paragraph are summarized in tabular form in Table S1.

Lastly, we can carry out the XX/attached-X test by comparing the relative success of Y-bearing sperm in XX dams (RS_Y(dams = XX)_ = {[M/E]_sire = skew_/[M/E]_sire = even_}_dam = XX_) to that with attached-X dams (RS_Y(dams = X∧X)_ = {[F/E]_sire = skew_/[F/E]_sire = even_}_dam = X∧X_). These two ratios should be the same assuming gametic drive is responsible for the female-biased sex ratio associated with the X_skew_/Y males, while with SA-zygotic drive ([M/E]_sire = skew_/[M/E]_sire = even_)_dam = XX_) should be smaller than ([F/E]_sire = skew_/[F/E]_sire = even_)_dam = X∧X_). To test the equality of these two ratios we constructed the metri
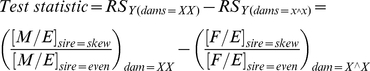
The observed value of the test statistic was −0.109 and its 95% upper-bound is −0.003, a value just less than zero, which is consistent with SA-zygotic drive but not gametic drive. This statistical test is significant (P<0.05, i.e., the 95% upper-bound does not overlap zero) despite an expected high sampling variance of its associated test statistic owing to the fact that it includes variation from four random variables simultaneously. To achieve statistical significance, despite this high expected variation, required us to trace the fate of a large sample of eggs (6,960 from X/X dams and 14,831 from attached-X dams).

In sum, three lines of evidence indicate that SA-zygotic drive, and not gametic drive alone, is responsible for the biased sex ratio produced by X_skew_/Y sires: *i)* a female-biased sex ratio when the dams were X/X but not male-biased when the dams were attached-X, *ii)* the sex ratio bias is associated with a depressed value of M/E, rather than an elevated value of F/E, compared to X_even_/Y sires (with the depressed value of M/E fully accounting for the observed sex ratio bias), and *iii)* an XX/attached-X test statistic that is significantly less than zero.

## Discussion

SA-zygotic drive is a phenomenon with potentially widespread ramifications that was earlier predicted to exist by population genetics theory [6], [7], [10]. Although there is correlative evidence that SA-zygotic drive may be operating in humans via grandparent-grandchild interactions [10], [15], no definitive examples in an experimentally tractable model organism had been documented. Identifying the exact mechanism that causes a biased sex ratio can be difficult, especially when the sex ratio at fertilization is unknown. Taking advantage of some of the genetic tools that have been developed for *Drosophila* model species, we designed a set of tests for SA-zygotic drive that accounts for gametic drive as well as potential viability and infertility effects, coded by the sex chromosome causing a sex-ratio bias. Using this test we extended the earlier work of Noor and Coyne [12], on an X-linked mutation (*skew^1^*) causing a female biased sex-ratio by an unknown mechanism. Our study on this mutation provides empirical support for the theoretical prediction that an X-linked son-killer phenotype can evolve and lead to SA-zygotic drive.

Our study does not represent irrefutable evidence for the operation of SA-zygotic drive because each of our three assays is not individually unambiguous. The predicted pattern of the relative magnitudes of M/E and F/E from X_skew_/Y and X_even_/Y sires when mated to XX dams (Fig. 2C) is only diagnostic of SA-zygotic drive when it can be shown that a false positive is not being produced by a fortuitous combination of gametic drive and pleiotropic effects of X_skew_ on the survival and fertility of its carriers. However, to obtain our observed pattern (M/E_skew_<M/E_even_≈F/E_even_≈F/E_skew_) via gametic drive, pleiotropic effects of X_skew_ would have to, by happenstance, nearly exactly counterbalance the level of gametic drive to produce the near equality of the observed values of M/E_even_, F/E_even_, and F/E_skew_. The finding in our second assay, of nearly identical sex ratios when X_skew_/Y and X_even_/Y sires were mated to attached-X dams, further supported the conclusion that SA-zygotic drive, rather than gametic drive, was responsible for the observed sex ratio imbalance. However, this negative result could have been fortuitously produced by gametic drive if the hemizygous effects of X_skew_ and X_even_ on viability precisely counterbalanced the sex ratio skew produced by gametic drive. Our last assay (XX/attached-X test), which fully controlled for any influence of X_skew_ and X_even_ on the survival and fertility (excluding dam-by-sire interactions) of its carriers, also indicated that the observed sex ratio bias associated with X_skew_/Y sires was due to SA-zygotic rather than gametic drive. This test could serendipitously produce a false negative for gametic drive if there was a sire-by-dam genotypic interaction for fertility that lowered the relative fertility of sperm from X_even_/Y sires to X_skew_/Y sires in attached-X dams. However, as can be seen from Fig. 3B, if anything sperm from X_skew_/Y sires had lower fertility than sperm from X_even_/Y sires with attached-X dams, making our test for SA-zygotic drive conservative. Another potential scenario that would make our XX/attached-X test produce a false negative for gametic drive would occur when the relative ability of X- and Y-bearing sperm to fertilize eggs differed between dams from the XX and attached-X stock in a way that exactly counterbalanced the effect of meiotic drive, and thereby equalized the sex ratio produced by the two types of sires. Despite all of these chance possibilities for false positives and negatives, we think that a strong case can be made that SA-zygotic drive is in operation in X_skew_/Y sires. We consider this conclusion to be robust because too many fortuitous, nearly-exact counterbalances would need to occur simultaneously by chance for our combined data set to so strongly support the operation of SA-zygotic drive. Finally our results could also have been influenced by the fact that the autosomal backgrounds carried by X_even_ and X_skew_ sires were not perfectly identical. Our data was collected over backcross generations 3 through 6 and the results for from each generation are presented in Fig. S1.

Ideally, we would carry out additional experiments to more fully document the SA-zygotic drive phenotype we describe here. However, the sex ratio phenotype in the skew population has not remained stable –precluding additional experimentation at this time. When Noor and Coyne [12] first assayed the sex ratio of the skew line in the early 1990's, it had 70% females (2.33♀∶1♂). Within two years, however, the sex ratio had declined to only 61% females (1.56♀∶1♂) and reduced egg hatch rate could fully account for the observed sex ratio bias. When we obtained the stock in 2008, the sex ratio was estimated to be 60% females (1.5♀∶1♂). Recently the sex ratio of the copy of this stock in our laboratory has increased to 66% (2♀∶1♂), there is no longer a sufficient excess in egg-to-adult mortality in broods from X_skew_/Y sires and XX dams to account for the observed sex ratio bias, and crosses of X_skew_/Y sires to attached-X dams results in a complete reversal of the sex ratio (66% males) with no extra egg-to adult mortality in broods from X_skew_/Y sires compared to X_even_/Y sires (data not shown). These recent observations suggest that the elevated sex ratio bias in the skew line is presently caused predominantly, or entirely, by gametic drive, and that transitions between gametic and SA-zygotic drive has recently evolved in our copy of the skew population. The stock with the *skew^1^* mutation has unfortunately recently been lost from the stock center, preventing new studies on the original population.

In flies, SA-zygotic drive can be mediated by *i)* sib-competition (harming opposite-sex siblings) and/or *ii)* trans-generational epigenetic effects (the X or Y epi-marks the opposite sex chromosome, or gender-specific genes expressed in the non-carrier sex, in a manner that causes miss-expression in the next generation). Because we reared flies at very low density, the sib-competition mechanisms seems least feasible. Both of the two epigenetic alternatives are feasible in our experiments, but the most parsimonious mechanism would be for the X to generically epi-mark the Y in a manner that disrupts its regulatory function in sons in the next generation. The “Winters” form of X-linked gametic drive in *D. simulans* causes the Y-bearing sperm to die during spermatogenesis [14]. This death of developing sperm is associated with a lack of condensation of the Y chromosome [16]: a phenotype feasibly produced via a trans-imprint from the X. SA-zygotic drive could be produced by different (or modified) trans-imprint on the Y that harmed diploid sons (rather than haploid sperm) due to changes in the way that the Y trans-regulates gene expression at non-linked loci, or possibly by disrupting decondensation of the Y during the first mitotic division.

Although imprinting is not well documented in *Drosophila* for the X and autosomes, except for their heterochromatic regions [17], there is clear evidence for parent-of-origin effects on the expression of the *Drosophila* Y chromosome [18], [19]. It has also been established that many regions of the *Drosophila* Y are capable of being imprinted and that imprinting is substantially more widespread on the Y than heterochromatic regions of the X and autosomes [20]. A recent microarray study has established that, despite its low content of structural genes [12 known genes: 21], the *Drosophila* Y chromosome regulates the activity of many hundreds of genes, at least in *D. melanogaster* [22]. These Y-trans-regulated genes tended to have male-restricted or male-biased (testes) expression [22], so a trans-imprint of the Y in the context of SA-zygotic drive could feasibly harms sons from XX dams more than daughters from attached-X dams. There is also recent evidence [19] that a paternal imprint of the Y chromosome can influence (down-regulate) the level of X-linked dosage compensation (expressed only in male *Drosophila*). Collectively these previous studies are consistent with a simple hypothesis for a mechanism causing the SA-zygotic drive that we have uncovered: The paternal X imprints the Y during spermatogenesis in a manner that disrupts the regulation of a critical ontogenetic pathway in developing embryos.

For an X-linked mutation to be able to spread through SA-zygotic drive, competition between daughters and sons has to be non-trivial [23], or males must harm their sisters due to sib-mating [24]. In natural populations of *D. melanogaster*, a close relative of *D. simulans* with similar breeding ecology, a study using electrophoretic markers indicated that it is common for only one or a few foundresses to contribute eggs to a single piece of rotting fruit [25]. Further support for the assumption that *D. simulans* has the requisite family structure to promote SA-zygotic drive comes from the observation that many *Drosophila* species in the *willistoni* group of fruit flies (that, like *D. simulans*, feed on fallen fruit) are infected with the male-killing intracellular bacteria *Spiroplasma* [26]. This male-killing bacterial strain has recently spread to *D. melanogaster* [27] and is now found on at least 3 continents. The fact that a male-killing bacteria has successfully spread to a close relatives to *D. simulans* (*D. melanogaster* with a similar fallen-fruit centered ecology), suggest that sib competition and/or harmful sib-mating in *D. simulans* also would be substantial enough to allow for a selfish element to spread through a male-killing phenotype. It should be noted, however, that SA-zygotic drive is more likely to evolve in species where close family associations are more pronounced than in *Drosophila*.

Our study provides the first experimental evidence that a new, and unappreciated, form of intragenomic conflict is operating in nature. This conflict is a simple consequence of the fact that, from an evolutionary perspective, X and Y chromosomes are intrinsic “mortal enemies” in the context of their transmission through competing offspring of opposite sex.

## Methods

To test for the operation of SA-zygotic drive by the X_skew_ chromosome studied by Noor and Coyne [12], we obtained the even (Florida City, stock number: 14021-0251.165) and skew (*garnet; cinnabar; ebony; skew;* stock number 14021-0251.093: g[1]; cn[1]; e[1];skew[1]) stocks from the Drosophila Species Stock Center (San Diego). Both stocks were weak, especially the skewed one, presumably due to accumulated inbreeding depression in the time since they were used by Noor and Coyne [12]. As a consequence, we backcrossed the X chromosomes from the skew and even stocks into a new genetic background. To keep the X chromosomes intact, we crossed males from the two lines to females carrying an attached-X (C(l)RM,*y w*/Y), and the resulting sons were repeatedly crossed to the attached-X females (taken anew from the attached-X stock) in each successive backcross generation. In this way we placed both X chromosomes into the same cytotype/Y/autosomes genetic background. Starting after 3 backcross generations (when 7/8^ths^ of the autosomal background had been replaced), we crossed X_skew_/Y and X_even_/Y males to females from the X_even_ stock and females from the attached-X stock. We used females from the X_even_ stock because Noor and Coyne [12] had demonstrated that the female-biased sex ratio of X_skew_/Y sires was manifest in this genetic background of dams. Males were mass mated to females (about 25 males with 25 females) by combining them in a single vial for 6–24 h. Following this mating period, the males were removed and then each female was placed in a separate vial with a narrow cut made in the cornmeal-molasses medium to induce egg-laying. After 20 h the females were removed and the eggs that had been laid were counted. Twelve days later, the number of male and female progeny were counted. To insure that each female had mated, only families from females that produced at least one surviving offspring were included in our sample. We then tallied, for each backcross generation separately (3, 4, and 5 for X/X dams and 3, 4, 5, and 6 for X∧X/Y dams), the total eggs, sons and daughters that were produced by all females, and calculated the proportion of total eggs that gave rise to adult sons and daughters. We then pooled data (counts) across all backcross generations. In the case of the crosses to attached-X dams, we extended our analysis by one generation in order to increase the total sample size (as these dams have lower fecundity and produce fewer viable offspring, compared to X/X dams). For matings to X/X dams, the number of families sampled in backcross generations 3, 4 and 5 was 31, 48, and 44, respectively, for X_skew_/Y sires, and 54, 41, and 51 for X_even_/Y sires. With these X/X dams, a total of 3,877 eggs (from 146 families) and 3,083 eggs (from 123 families) were screened for X_even_/Y and X_skew_/Y sires, respectively. With attached-X dams, the number of families sampled in backcross generations 3, 4, 5 and 6 was 41, 32, 70, and 168, respectively, for X_skew_/Y sires, and 42, 56, 80, and 219, respectively, for X_even_/Y sires. With these attached-X dams a total of 8,270 (from 397 families) and 6,561 eggs (from 311 families) were screened for X_even_/Y and X_skew_/Y sires, respectively. All confidence intervals reported below were obtained by bootstrapping the data (using individual families as the unit for resampling, and resampling data from each backcross generation separately). Bootstrapping was carried out with the *Statistics-101* public domain resampling program using 50,000 bootstraps for each estimate.

## Supporting Information

Figure S1 **A plot of F/E (red) and M/E (Blue) by backcross generation (3–6) and sire X chromosome (FC or SK) with XX dams and attached-X dams.**
(PDF)

Table S1 Sample means and bootstrap 95% confidence intervals for M/E and F/E.
(DOCX)

Appendix S1 Description the previous study by Noor and Coyne on the *skew* mutation.
(DOC)

Appendix S2 Predictions of *RS_Y(dams = XX)_* and *RS_Y(dams = X∧X)_* when gametic drive is operating.
(DOC)

Appendix S3 Predictions *RS_Y(dams = XX)_* and *RS_Y(dams = X∧X)_* when SA-zygotic drive is operating.
(DOCX)
