# Supplementary material for: Empirical Evidence for Son-Killing X Chromosomes and the Operation of SA-Zygotic Drive
Source: PLoS One. 2011 Aug 17;6(8):e23508. doi: 10.1371/journal.pone.0023508 (PMC3157394; doi:10.1371/journal.pone.0023508)
Supplement: Figure S1 — A plot of F/E (red) and M/E (Blue) by backcross generation (3–6) and sire X chromosome (FC or SK) with XX dams and attached-X dams. (PDF) [file pone.0023508.s001.pdf]

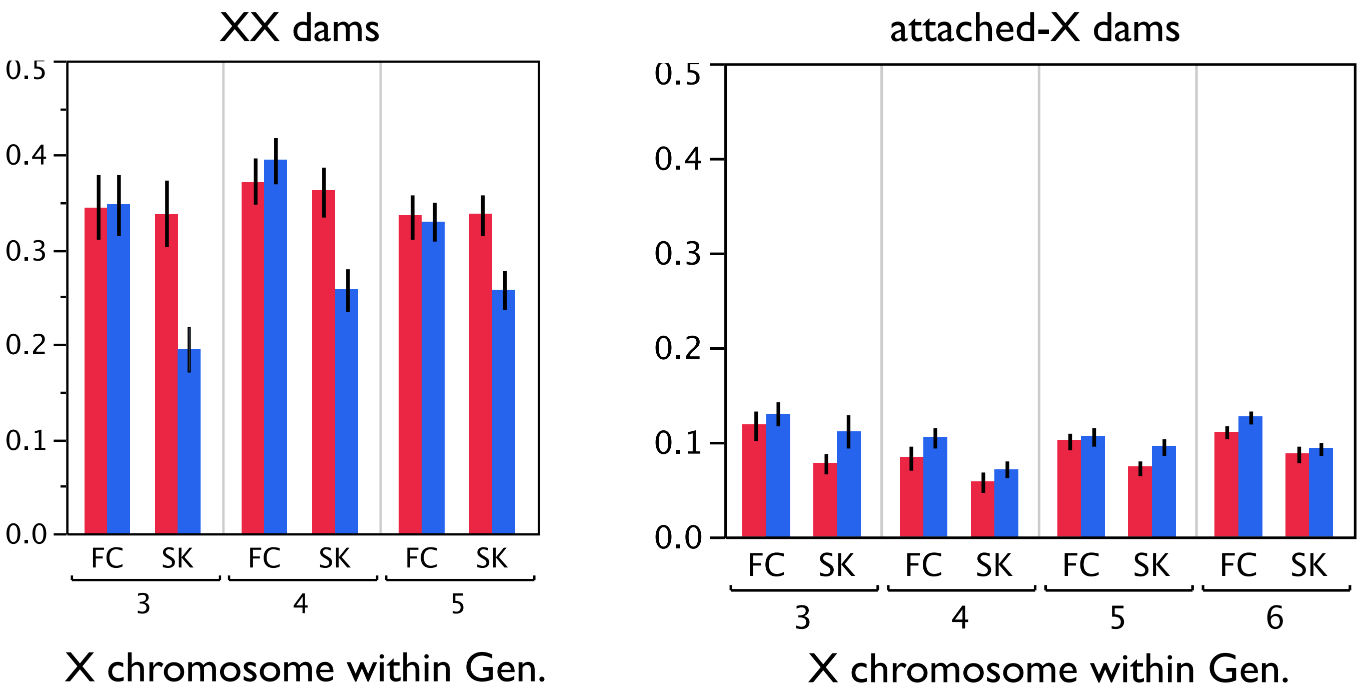

Figure S1. A plot of F/E (red) and M/E (Blue) by backcross generation (3-6) and sire X chromosome (FC or SK) with XX dams and attached-X dams.
