# Supplementary material for: Empirical Evidence for Son-Killing X Chromosomes and the Operation of SA-Zygotic Drive
Source: PLoS One. 2011 Aug 17;6(8):e23508. doi: 10.1371/journal.pone.0023508 (PMC3157394; doi:10.1371/journal.pone.0023508)
Supplement: Table S1 — Sample means and bootstrap 95% confidence intervals for M/E and F/E. (DOCX) [file pone.0023508.s002.docx]

**Table S1**

Table 1. Sample means and bootstrap 95% confidence intervals for M/E and F/E.

|  | **Dam(X/X)** | | **Dam(X^X/Y)** | |
| --- | --- | --- | --- | --- |
|  | **Sire(X_even_/Y)** | **Sire(X_skew_/Y)** | **Sire(X_even_/Y)** | **Sire(X_skew_/Y)** |
| F/E: | 0.351 | 0.343 | 0.106 | 0.081 |
| CI_F/E_: | (0.321, 0.380) | (0.310, 0.376) | (0.098, 0.115) | (0.074, 0.088) |
| M/E: | 0.357 | 0.233 | 0.121 | 0.094 |
| CI_M/E_: | (0.329, 0.385) | (0.208, 0.258) | (0.112, 0.129) | (0.084, 0.103) |
