# Supplementary material for: Empirical Evidence for Son-Killing X Chromosomes and the Operation of SA-Zygotic Drive
Source: PLoS One. 2011 Aug 17;6(8):e23508. doi: 10.1371/journal.pone.0023508 (PMC3157394; doi:10.1371/journal.pone.0023508)
Supplement: Appendix S1 — Description the previous study by Noor and Coyne on the skew mutation. (DOC) [file pone.0023508.s003.doc]

**Appendix S1**

**The Noor and Coyne study**

Noor and Coyne (1995) observed a strongly female-biased sex ratio in a line that had each major chromosome marked with a visible mutation. Such a line probably placed the X in a new genetic background because each chromosomal marker likely originated in a different stock population. Reciprocal crosses were made between the skewed sex ratio line (hereafter called ‘skew’) and an unmarked line that had an even sex ratio (an isofemale line originating in Florida City, FL, USA –hereafter called ‘even’). In combination with backcrosses, these data showed that the biased sex ratio was associated with the X chromosome from the skew line, but only when it was expressed in males. The X-linked factor coding for the female-biased sex ratio was mapped to the proximal tip of the X chromosome. The biased sex ratio phenotype was also shown to be unaffected by the cytoplasm of mothers (skew vs. even), rearing temperature (18º vs. 23º C) and treatment with antibiotics (tetracycline). These data were consistent with an X-linked gametic drive element that operated in fathers. But an alternative explanation was also possible: mortality or increased survival that is associated with the expression of X-linked genes can also lead to biased sex ratios. A strong case for gametic drive can be made, however, when the total egg-to-adult mortality is too small to account for the observed sex ratio bias. Evidence against gametic drive occurs when there is elevated mortality in families from the cross exhibiting a biased sex ratio, and this extra mortality, when assigned to the rarer sex, can account for the biased sex ratio. When Noor and Coyne measured the hatch rate of eggs from families sired by males that were isogenic except for the presence or absence of the skew X chromosome (X_skew_), they reported that “*the reduction in egg hatch could account for the sex ratio bias, and we cannot conclude that the skew resulted from meiotic drive.*” This is a disappointing result for researchers searching for gametic drive and, as a consequence, Noor and Coyne terminated their study in response to this finding and deposited the skew and even stocks in an international *Drosophila* stock center. From the perspective of SA-zygotic drive, however, Noor and Coyne’s results were suggestive that this process may be operating in *D. simulans*.

To interpret Noor and Coyne’s results from the perspective of SA-Zygotic drive, it is useful to quantitatively compare egg-to-adult survival to the observed sex ratio bias. At the end of their study, the sex ratio of families from X_skew_/Y sires had declined to 61% females (from 70% at the start of their study), while that from X_even_/Y sires was unbiased (reported to be 50.1% females at the start of their study). In these assays, males were produced from reciprocal crosses between the even and skew stocks and then sex ratios were measured when the two types of sires were mated to females from the even stock. Also at the end of their study, Noor and Coyne reported the egg hatch rate to be 91.6% for families sired by X_skew_/Y males and 76% for those sired by X_even_/Y males. This account almost certainly mistakenly reversed the ‘percentage egg hatch’ and ‘cross categories’ since two sentences later in the same paragraph the authors stated that “*the female biased sex ratio was associated with a reduced hatch rate*” and in the next paragraph “*Thus, the cause of the skew in the sex ratio is unknown, but it is associated with increased egg lethality.*” The first author also agreed with our interpretation when we recently contacted him (M. Noor, personal communication). Correcting this presumed typographical error, and assuming that the lower egg hatch rate associated with families sired by X_skew_ males accounts for essentially all egg-to-adult mortality, the percent of fertilized eggs becoming adult males and adult females would be:

X_even_ sires: 45.9% ♀ and 45.7% ♂

X_skew_ sires: 46.4% ♀ and 29.6% ♂

Note the low proportion of the original cohort of eggs fertilized by X_skew_/Y sires that survived and became adult sons, and the consistently higher and similar values of this metric for the other three sire/sex-of-offspring dyads. Noor and Coyne stated that they conducted their egg-hatch assay because “*The sex ratio bias could result from either a meiotically-driven X-chromosome, or a form of greater mortality of males possessing this X-chromosome*.” However, their results cannot be explained by mortality associated with males possessing X_skew_ because sons from X_skew_/Y sires do not carry their father’s X_skew_ chromosome. Their results are consistent, however, with a female-biased sex ratio due to SA-zygotic drive, i.e., increased mortality of sons of X_skew_/Y sires (that do not carry his X chromosome), but not of daughters (that do express their father’s X), can account for the sex ratio bias observed by Noor and Coyne (1995). Such SA-zygotic drive could have been mediated by a paternal effect that harmed sons (that do not carry the father’s X chromosome). However, because Noor and Coyne did not measure total egg-to-adult survival (only hatch rate), and because gametic drive, when combined with lower fertility of X_skew_/Y sires and/or lower survival of X/X_skew_ daughters, can also produce the observed pattern (see fig 2 main text), additional experiments with suitable measurements of egg-to-adult mortality were needed to demonstrate the operation of SA-zygotic drive in this model organism.

**References**

Noor MAF, Coyne JA (1995) Research Note. Drosophila information service 76: 151-152.
