# Supplementary material for: Empirical Evidence for Son-Killing X Chromosomes and the Operation of SA-Zygotic Drive
Source: PLoS One. 2011 Aug 17;6(8):e23508. doi: 10.1371/journal.pone.0023508 (PMC3157394; doi:10.1371/journal.pone.0023508)
Supplement: Appendix S2 — Predictions of RSY(dams = XX) and RSY(dams = X∧X) when gametic drive is operating. (DOC) [file pone.0023508.s004.doc]

**Appendix S2**

***RS_Y(dams=XX)_* and *RS_Y(dams=X^X)_* when gametic drive is operating**

Assume that X_skew_ produces a female biased adult sex ratio by gametic drive, i.e. fewer Y-bearing sperm fertilize eggs. When X_even_/Y and X_skew_/Y sires are crossed to normal X/X dams, their sons, on average, have the same genome-wide genotypes and maternal effects, so average egg-to-adult survival of sons from these crosses should be identical, barring any paternal effect on his offspring’s survival. More specifically, the proportion of total eggs (E) that give rise to eclosing sons [M/E] when the sire is X_even_/Y is:

[M/E]_sire=even_ = ½*Fertility_sire=even_*E_X/X_*survival_XY_*Paternal_sire=even_,

Where E_X/X_ is the proportion of viable eggs produced by X/X dams, Paternal_sire=even_ is any potential paternal effect on the viability of offspring (of both sexes) sired by Y/X_even_ males and Fertility_sire_ is the proportion of dams eggs that are fertilized by a sire. In contrast, the proportion of eggs that give rise to eclosing sons [M/E] when the sire is X_skew_/Y is:

[M/E]_sire=skew_ = (½*MD)* Fertility_sire=skew_* E_X/X_ *survival_XY_*Paternal_sire=skew_,

where MD is a scalar (<1) measuring the intensity of gametic drive.

We next compare the relative success of Y-bearing sperm (*RS_Y_*) from the two types of sires:

[M/E]_sire=skew_ (½*MD)*E_X/X_*Fertility_sire=skew_*survival_XY_*Paternal_sire=skew_

*RS_Y(dams=XX)_* = ^_________________^ = ^_____________________________________________^

. [M/E]_sire=even_ ½*E_X/X_*Fertility_sire=even_*survival_XY_*Paternal_sire=even_.

= MD* (Fertility_sire=skew_ /Fertility_sire=even_)*(Paternal_sire=skew_/Paternal_sire=even_) (1)

The metric *RS_Y_* is proportional to the fraction of fertilizations by Y-bearing sperm by the two types of sires. It depends not only on the strength of gametic drive (MD), but also on the relative fertility and paternal effects (affecting both sexes) of X_skew_/Y and X_even_/Y sires. To eliminate the latter two confounding factors we need to mate the males to attached-X dams.

When the dams are attached-X (X^X/Y), sires of both types produce daughters (from Y-bearing sperm, Fig. 1) with, on average, the same genome-wide genotypes and maternal effects. Daughters from both types of sires are therefore expected to have identical average survival, unless there is a paternal effect influencing daughter-viability.

The proportion of eggs that give rise to eclosing daughters [F/E] when the sire is X_even_/Y is:

[F/E]_sire=even_ = ¼*Fertility_sire=even_*E_X^X/Y_*survival_X^X/Y_*Paternal_sire=even_.

Note the value of ¼ replaces ½ in the above equations because half of all embryos die due to aneuploid genomes (X/X^X or YY). The proportion of eggs that give rise to eclosing daughters [F/E] when the sire is X_skew_/Y is:

[F/E]_sire=skew_ = (¼*MD) *Fertility_sire=skew_*E_X^X/Y_ *survival_X^X/Y_*Paternal_sire=skew_.

We again compare the relative success of Y-bearing sperm (*RS_Y_*) from the two types of sires, but in this case with attached-X dams:

[F/E]_sire=skew_ (¼*MD)*E_X^X/Y_*Fertility_sire=skew_*survival_X^X/Y_*Paternal_sire=skew_

*RS_Y(dams=X^X)_* = ^_________________^ = ^________________________________________^

. [F/E]_sire=even_  ¼* E_X^X/Y_*Fertility_sire=even_*survival_X^X/Y_*Paternal_sire=even_.

= MD* (Fertility_sire=skew_ /Fertility_sire=even_)*(Paternal_sire=skew_/Paternal_sire=even_) (2)

With attached-X dams, the metric *RS_Y_* is also proportional to the fraction of fertilizations by Y-bearing sperm by the two types of sires, and depends on the strength of gametic drive (MD) as well as the relative fertility and paternal effects (affecting both sexes) of X_skew_/Y and X_even_/Y sires –but the latter two confounding factors are the same with both types of dams. Comparing equations (1) and (2) demonstrates that

[M/E]_sire=skew_ [F/E]_sire=skew_

^_________________^ = ^________________^ or *RS_Y(dams=XX)_* = *RS_Y(dams=X^X)_*

[M/E]_sire=even_ [F/E]_sire=even_

when gametic drive is operating and the M/E data come from X/X dams and the F/E data from X^X dams. Deviations from this equality provide evidence against the operation of gametic drive (as the sole agent, excluding SA-zygotic drive, causing a female-biased sex ratio) that is robust to any influence of X_skew_ on a sire’s fertility and paternal effects, and any viability effects in offspring carrying it. This approach, however, assumes that fertility of the two types of sires is constant across two types of dams, as discussed in the main body of the text.
