# Supplementary material for: Empirical Evidence for Son-Killing X Chromosomes and the Operation of SA-Zygotic Drive
Source: PLoS One. 2011 Aug 17;6(8):e23508. doi: 10.1371/journal.pone.0023508 (PMC3157394; doi:10.1371/journal.pone.0023508)
Supplement: Appendix S3 — Predictions RSY(dams = XX) and RSY(dams = X∧X) when SA-zygotic drive is operating. (DOCX) [file pone.0023508.s005.docx]

**Appendix S3**

***RS_Y(dams=XX)_* and *RS_Y(dams=X^X)_* when SA-zygotic drive is operating**

First we derive the ratios of males per eggs when fathers are mated to X/X females:

[M/E]_sire=even_ = ½* E_XX_*Fertility_sire=even_* survival_XY_*Paternal_sire=even_

[M/E]_sire=skew_ = (½*SAZD)*E_XX_*Fertility_sire=skew_*survival_XY_*Paternal_sire=skew_,

where SAZD is a scalar (<1) measuring the strength of SA-zygotic drive, and all other terms are as defined in the main manuscript. The expected ratio of [M/E] from the two crosses (*RS_Y_*, measuring the relative reproductive success of Y-bearing sperm from the two types of sires) is therefore:

[M/E]_sire=skew_ (½*SAZD) E_XX_**Fertility_sire=skew_*survival_XY_*Paternal_sire=skew_

*RS_Y(dams=XX)_*  = ^_________________^ = ^__________________________________________________________^

[M/E]_sire=even_ ½* E_XX_*Fertility_sire=even_* survival_XY_ *Paternal_sire=even_

= SAZD* (Fertility_sire=skew_ / Fertility_sire=even_)*(Paternal_sire=skew_/Paternal_sire=even_) (3)

Next we derive the ratios of females per eggs when father are mated to attached-X females.

[F/E]_sire=even_ = ¼*E_X^X/Y_*Fertility_sire=even_*survival_X^X/Y_*Paternal_sire=even_

[F/E]_sire=skew_ = ¼*E_X^X/Y_*Fertility_sire=skew_*survival_X^X/Y_*Paternal_sire=skew_.

Note that the equation for [F/E]_sire=skew_ assumes that SA-zygotic drive does not operate through a an X-coded imprint on the Y that disrupts an ontogenetic pathway in both sexes of offspring.

The expected ratio of [F/E] from the two crosses (*RS_Y_*, measuring the relative reproductive success of Y-bearing sperm from the two types of sires) is therefore:

[F/E]_sire=skew_ ¼*E_X^X/Y_*Fertility_ssire=skew_*survival_X^X/Y_*Paternal_sire=skew_

*RS_Y(dams=X^X)_*  = ^_________________^ = ^______________________________________________________________^

[F/E]_sire=even_ ¼*E_X^X/Y_*Fertility_sire=even_*survival_X^X/Y_*Paternal_sire=even_

= (Fertility_sire=skew_ / Fertility_sire=even_)*(Paternal_sire=skew_/Paternal_sire=even_) (4)

Comparing equations (3) and (4) demonstrates that

[M/E]_sire=skew_ [F/E]_sire=skew_

^_________________^ < ^________________^ or *RS_Y(dams=XX)_* < *RS_Y(dams=X^X)_*

_._ [M/E]_sire=even_ [F/E]_sire=even_

when SA-zygotic drive is operating.
